# Supplementary material for: Prognostic Immune Effector Signature in Adult Acute Lymphoblastic Leukemia Patients Is Dominated by γδ T Cells
Source: Cells. 2023 Jun 22;12(13):1693. doi: 10.3390/cells12131693 (PMC10340700; doi:10.3390/cells12131693)
Supplement: Supplementary file 1 [file cells-12-01693-s001.zip › cells-2325612-supplementary.pdf]

Supplementary Materials

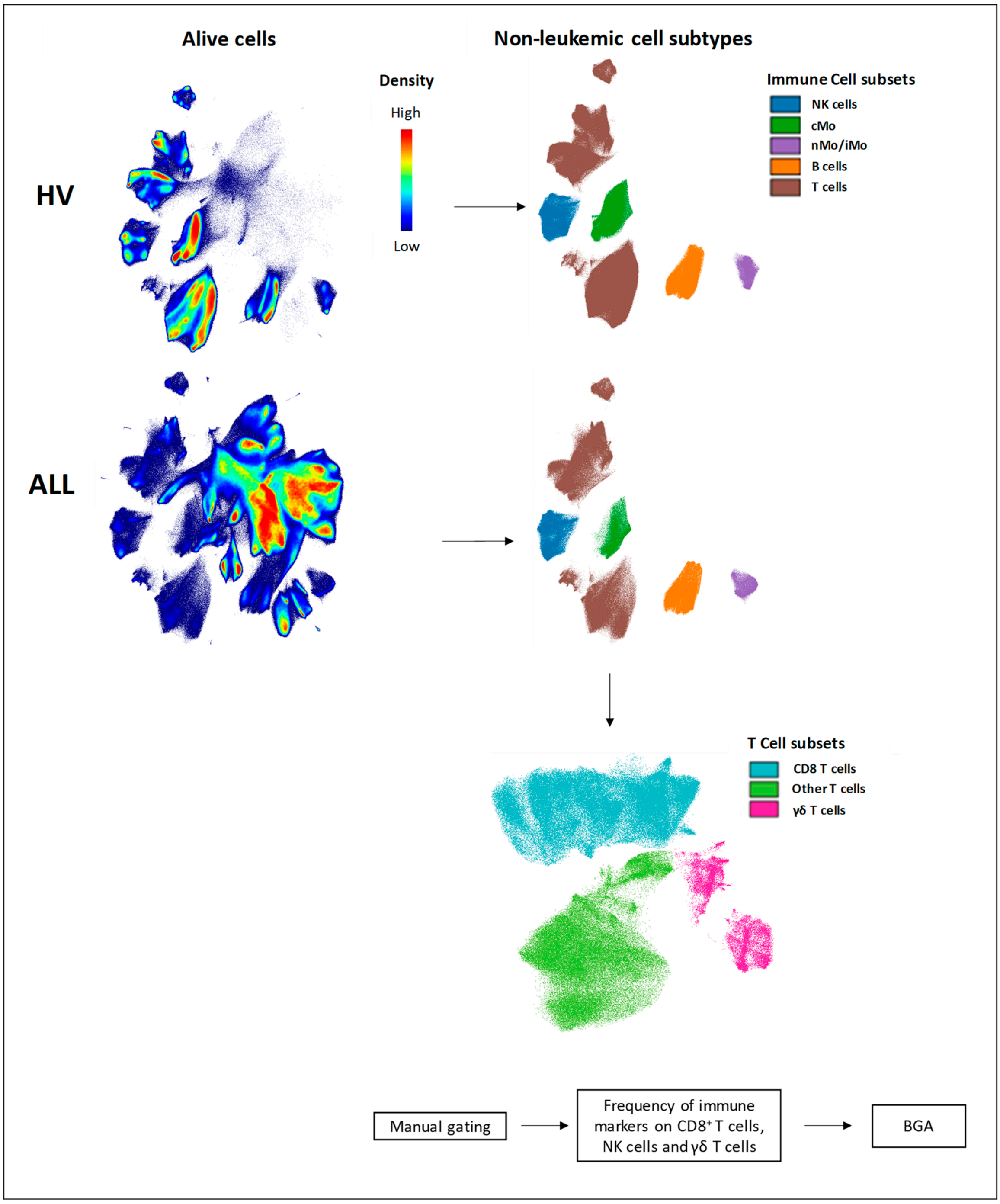

Figure S1. Dimensionality-reduction analysis of PBMCs from HV and newly diagnosed ALL patients enables identification of non-leukemic cells. Consensus files were generated using uniform manifold approximation and projection (UMAP) dimensionality-reduction technique. Leukemic cells were excluded from a total of 4,800,000 alive cells (1,200,000 cells in each group; HV, B-ALL, T-ALL and Ph^+ ALL). Immune cell subsets and T-cell subsets from HV and ALL patients were preliminary identified from consensus files generated using Uniform Manifold Approximation and Projection (UMAP) dimensionality-reduction technique. Frequency of immune markers were then manually gated and used in the BGA.

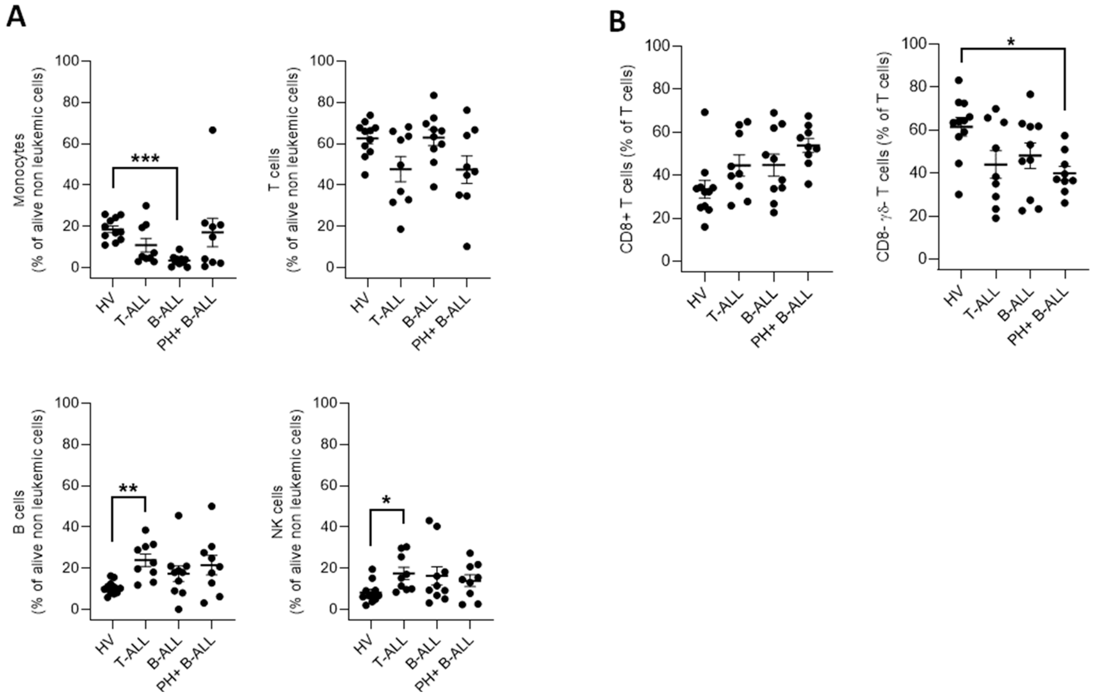

Figure S2. Comparative frequencies of circulating immune subsets in HV and ALL patients. Proportion of immune subset in PBMCs of 11 HV, 10 B-ALL, 9 B-ALL Ph^{+} and 9 T-ALL patients. (**A**) Proportion of monocytes, T cells, B cells and NK cells among non-leukemic cells (%). (**B**) Proportion of $CD8^{+}$ T cells and $CD8^{-}γδ^{-}$ T cells among T cells (%). Data are expressed as mean ± SEM. The statistical significance was established using a Kruskall-Wallis with Dunn's post-test. *p < 0.05.

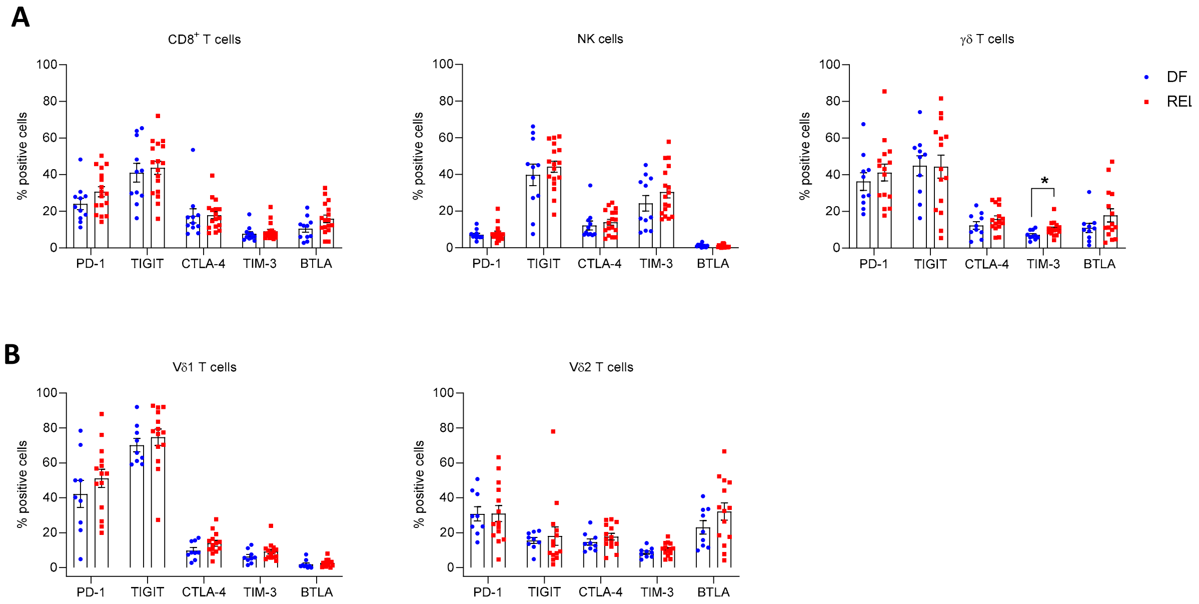

Figure S3. Immune check-point expression on circulating immune effectors. Comparison of the expression of PD-1, TIGIT, CTLA-4, TIM-3 and BTLA expression (**A)** on the surface of CD8^{+} T cells and NK cells (n=28 patients), ɣδ T cells (n=25 patients); (**B)** on the surface of Vδ1 T cells and Vδ2 T cells (n=23 patients). Data are expressed as mean ± SEM. The statistical significance was established using a Mann-Whitney test. *$p< 0.05$.

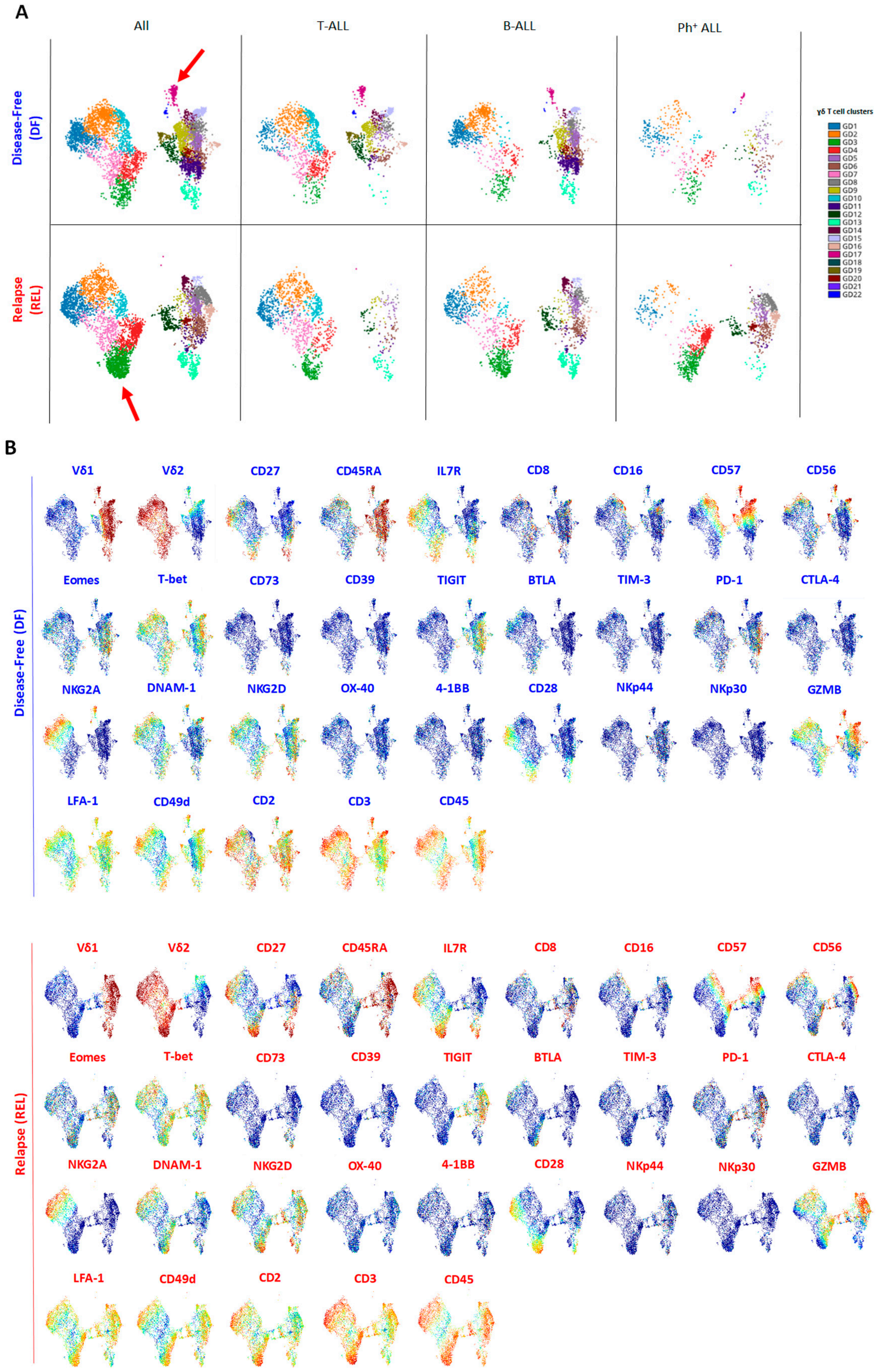
A
All
T-ALL
B-ALL
Ph+ ALL
Disease-Free (DF)
Relapse (REL)
γδ T cell clusters
GD1
GD2
GD3
GD4
GD5
GD6
GD7
GD8
GD9
GD10
GD11
GD12
GD13
GD14
GD15
GD16
GD17
GD18
GD19
GD20
GD21
GD22
B
Disease-Free (DF)
Vδ1 Vδ2 CD27 CD45RA IL7R CD8 CD16 CD57 CD56
Eomes T-bet CD73 CD39 TIGIT BTLA TIM-3 PD-1 CTLA-4
NKG2A DNAM-1 NKG2D OX-40 4-1BB CD28 NKp44 NKp30 GZMB
LFA-1 CD49d CD2 CD3 CD45
Relapse (REL)
Vδ1 Vδ2 CD27 CD45RA IL7R CD8 CD16 CD57 CD56
Eomes T-bet CD73 CD39 TIGIT BTLA TIM-3 PD-1 CTLA-4
NKG2A DNAM-1 NKG2D OX-40 4-1BB CD28 NKp44 NKp30 GZMB
LFA-1 CD49d CD2 CD3 CD45

Figure S4. Vδ1 and Vδ2 T-cell clusters have different repartition according to ALL lineage. (**A**) UMAP and PhenoGraph clustering allows the automatic identification of 22 γδ T-cell clusters according to the coexpression of 32 markers. Upper panels concern DF group and from right to left are described all DF patients, T-ALL, B-ALL and Ph^+ ALL patients (n=4; 4 and 3 respectively). Lower panels concern REL group and from right to left are described all REL patients, T-ALL, B-ALL and Ph^+ ALL patients (n=5; 6 and 6 respectively). The red arrows in the right panels indicate γδ T-cell clusters with similar changes according to the relapse group. (**B**) Expression of γδ T-cell markers which were used for PhenoGraph clustering (blue, low expression; red, high expression).

Supplementary Table S1. Patient, disease characteristics and response to treatment

	All patients<br>N = 28 (100)
Age, years	
Median [range]	48 [23-80]
>60	8 (28.6)
Gender	
Male	14 (50)
Female	14 (50)
WBC, x10^9/L	
Median [range]	76.1 [0.1-347]
High WBC count	19 (67.8)
FAB subtype	
B	19 (67.9)
B Ph-	10 (52.6)
B Ph+	9 (47.4)
T	9 (32.1)
PB blasts, %	
Mean (SD)	84.3 (9.6)
Induction	28 (100)
CR after induction	100 (100)
MRD status	
MRD+	13 (46.4)
MRD-	9 (32.1)
NA	6 (21.5)
Allo-SCT	14 (50)
Relapse	17 (60.7)

Allo-SCT: allogenic hematopoietic stem cell transplantation in 1st complete remission; CR: complete remission; High WBC count : ≥ 30 *10^9/L for B lineage and ≥ 100* 10^9/L for T lineage; MRD : Minimal Residual Disease; NA : Not available; Ph-: Philadelphia chromosome-negative ALL; Ph+: Philadelphia chromosome-positive ALL; PB : peripheral blood; WBC: white blood cells.

Supplementary Table S2. Mass cytometry panel — Extracellular antibodies

Antibodies	Metal	Clones	Source
CD45	89Y	HI30	Fluidigm
CD2	106Cd	39c1.5	Immunomonitoring Lab
LFA1	110Cd	2H8.3	Immunomonitoring Lab
CD19	111Cd	HIB19	Purified : BioLegend and metal labeling kit : Fluidigm
CD33	112Cd	WM53	Purified : BioLegend and metal labeling kit: Fluidigm
Vδ2	114Cd	IMMU389	Purified : Beckman Coulter and metal labeling kit: Fluidigm
CD3	115In	UCHT1	Immunomonitoring Lab
CD57	116Cd	HNK-1	Purified : BioLegend and metal labeling kit: Fluidigm
BTN3A	141Pr	20.1	Purified : Imcheck Therapeutic and metal labeling kit: Fluidigm
CD45RA	143Nd	HI100	Fluidigm
CD73	145Nd	AD2	Purified : BioLegend and metal labeling kit: Fluidigm
CD8α	146Nd	RPA-T8	Fluidigm
OX40	150Nd	ACT35	Fluidigm
BTN2A1	151Eu	7.48	Purified : Imcheck Therapeutic and metal labeling kit: Fluidigm
CD39	152Sm	A1	Purified : BioLegend and metal labeling kit: Fluidigm
TIM-3	153Eu	F38-2E2	Fluidigm
TIGIT	154Sm	MBSA43	Fluidigm
PD-1	155Gd	EH12-2H7	Fluidigm
4-1BB	158Gd	4B4-1	Fluidigm
CD28	160Gd	CD28.2	Fluidigm
BTLA	163Dy	MIH26	Fluidigm
NKG2A	165Ho	REA110	Purified : Miltenyi and metal labeling kit: Fluidigm
NKG2D	166Er	ON72	Fluidigm
CD27	167Er	O323	Fluidigm
IL-7R	168Er	A019D5	Fluidigm
NKp30	169Tm	Z25	Purified :Beckman Coulter and metal labeling kit: Fluidigm
TCRpanγδ	170Er	REA591	Purified : Miltenyi and metal labeling kit: Fluidigm
DNAM-1	171Yb	DX11	Fluidigm
Vδ1	172Yb	REA173	Purified : Miltenyi and metal labeling kit: Fluidigm
ITGA4/CD49d	174Yb	9F10	Fluidigm
CD56	176Yb	NCAM16.2	Fluidigm
NKp44	159Tb	P44-8	Purified : Biolegend and metal labeling kit: Fluidigm
CD16	209Bi	3G8	Fluidigm

Mass cytometry panel — Intracellular antibodies

Antibodies	Metal		Source
Eomes	149Sm	X4-83	Purified : BD Biosciences and metal labeling kit: Fluidigm
Tbet	156Gd	4B10	Fluidigm
CTLA-4	161Dy	14D3	Fluidigm
Granzyme B	173Yb	GB11	Fluidigm
BTN2A2	175Lu	418A7612	Purified : ImCheck Therapeutics and metal labeling kit: Fluidigm
