# Supplementary material for: Prognostic Immune Effector Signature in Adult Acute Lymphoblastic Leukemia Patients Is Dominated by γδ T Cells
Source: Cells. 2023 Jun 22;12(13):1693. doi: 10.3390/cells12131693 (PMC10340700; doi:10.3390/cells12131693)
Supplement: Supplementary file 1 [file cells-12-01693-s001.zip › Supplementary File S1.pdf]

```
library(made4)

library(ggplot2)

D <- data.frame(read.csv2("File location",dec=".", sep=";"))

mark_type = c(rep("GD.",32),rep("NK.",29),rep("CD8.",28))

DAT <- D[which( D$RELAPSE == "AR" | D$RELAPSE == "NO") ,]

nrow(DAT)

mat <- DAT[,colnames(DAT) != "NK.count" & colnames(DAT) != "CD8.count"

        & colnames(DAT) != "GD.count" & colnames (DAT) != "RELAPSE"

        & colnames(DAT) != "ID" & colnames(DAT) != "PHENO"

        & colnames(DAT) != "file"]

dim(mat)

# plot the BGA

as.numeric(mat)

group <- DAT$RELAPSE

res.bga = bga(t(mat), factor(group))

plot(res.bga)

gene <- topgenes(res.bga, axis = 1)

gene <- topgenes(res.bga, axis = 2, end="neg",n=10)

marker <- data.frame(mark = colnames(mat), mark_type = mark_type)

n_mark <- data.frame(type = c("GD.","NK.","CD8."),n_mark = c(32,29,28))

col_coord <- res.bga$bet$co

df_contrib <- data.frame(var = colnames(mat), contribution = col_coord$Comp1) #comp1 = axe1

df_contrib <- cbind(df_contrib,marker)

tot_contrib_pos = sum(df_contrib[df_contrib$contribution > 0,]$contribution)

tot_contrib_neg = sum(df_contrib[df_contrib$contribution < 0,]$contribution)

df_contrib$percent_contrib = ifelse(df_contrib$contribution <0,
df_contrib$contribution/tot_contrib_neg*100,

                              df_contrib$contribution/tot_contrib_pos*100)

df_contrib <- df_contrib[order(df_contrib$contribution,decreasing = TRUE), ]

head(df_contrib)

tail(df_contrib)
```

```
n_mark = nrow(df_contrib)

df_contrib$num <- as.factor(c(1:n_mark))

df_contrib$percent_contrib_signed <- ifelse(df_contrib$contribution > 0,
df_contrib$percent_contrib,-df_contrib$percent_contrib)

write.csv2(df_contrib,"I:/Cytof_ALL2/df_contribGLOBAL1.csv",row.names=FALSE)
```
